# First description of the worker caste of *Nylanderia smythiesii* (Hymenoptera: Formicidae)

**DOI:** 10.3897/BDJ.2.e1163

**Published:** 2014-08-12

**Authors:** Aijaz Ahmad Wachkoo, Himender Bharti

**Affiliations:** † Punjabi University, Patiala, India

**Keywords:** Formicinae, Himalaya, *species inquirenda*, key, ants, taxonomy

## Abstract

The hitherto unknown worker caste of *Nylanderia smythiesii* (Forel, 1894) is described for the first time. Sexuals are redescribed and photomontage images of all castes are provided. A key is presented to separate the six Indian species of *Nylanderia*. Previously described *Nylanderia assimilis* (Jerdon, 1851) is considered a *species inquirenda*.

## Introduction

Based on the taxonomy and molecular phylogeny of *Prenolepis* genus-group LaPolla et al. (2010) raised *Nylanderia*, a formerly synonymized subgenus to generic status. It currently includes 108 extant species, 26 subspecies and 2 fossil species, distributed worldwide, but reaches its highest diversity in tropics (LaPolla et al. 2010, AntWeb 2014b). In India, prior to this study *Nylanderia* was represented by eight species (AntWeb 2014a).

LaPolla et al. (2011a) discussed the systematics and biology of *Nylanderia* in a global context. As a part of world monographic series addressing the species-level taxonomy of the ant genus *Nylanderia*, LaPolla et al. (2011b) revised this genus from Afrotropics whereas Kallal and LaPolla (2012) revised the Nearctic *Nylanderia*. While the majority of regions are awaiting taxonomic revision, most of the work prior to 2010 is under *Paratrechina*. Significant taxonomic contributions to this genus from Southeast Asia include, Morisita et al. (1991), Wu and Wang (1995), Terayama (1999), Zhou (2001). In India, Bingham (1903) is the only taxonomic work on *Nylanderia*.

Here we describe the hitherto unknown worker caste of *Nylanderia smythiesii* (Forel, 1894) collected in foothills of Northwest Himalaya, the Shivalik range. Due to inadequate description and unavailability of type material, status of *Nylanderia assimilis* (Jerdon, 1851) seems doubtful and is considered a *species inquirenda* to minimize confusion by eliminating future use of this name. We also, provide a key to known species of *Nylanderia* from India; however, *Nylanderia aseta* (Forel, 1902) is excluded from the key as it will be transferred to *Paraparatrechina* (Bharti and Wachkoo, in prep.).

## Materials and methods

The specimens were collected through Winkler’s extractor, pitfall, honey bait, beating vegetation, soil core and hand picking methods. The taxonomic study was conducted on a Nikon SMZ 1500 stereomicroscope. For digital images, MP evolution digital camera was used on the same microscope with Auto-Montage (Syncroscopy, Division of Synoptics, Ltd.) software. Later, images were cleaned with Adobe Photoshop CS5. Specimens are deposited in PUPAC, Punjabi University Patiala Ant Collection, Patiala. Some worker specimens will be deposited in BMNH, Natural History Museum, London, U.K. and CASC, California Academy of Sciences, San Francisco, United States of America. Morphological terminology for measurements (given in millimeters) and indices include:

HL – Maximum length of head in full-face view, measured in straight line from the anterior most point of the median clypeal margin to a line drawn across the posterior margin from its highest points (to accommodate species where the posterior margin is concave).

HW – Maximum width of head in full-face view (excluding the portion of eyes that extends past the lateral margins of the head).

EL – Maximum length of eye as measured normally in oblique view of the head to show full surface of eye.

SL – Maximum length of the scape excluding the basal neck and condyle.

PW – Maximum width of the pronotum in dorsal view.

WL – Weber’s length measured from the anterior surface of the pronotum proper (excluding the collar) to the posteriormost point of the propodeal lobes.

PrFL – Maximum length of the profemur from its margin with the trochanter to its margin with the tibia.

PrFW – Maximum width of the profemur.

CI – Cephalic index: HW/HL × 100.

SI – Scape index: SL/HW × 100.

REL – Relative eye length index: EL/HL × 100.

## Taxon treatments

### Nylanderia smythiesii

(Forel, 1894)

Prenolepis smythiesii  Forel, 1894 – Forel 1894: 410, fig. 5 (q.m.) INDIA. Combination in Paratrechina (Nylanderia): Emery 1925: 220; in *Nylanderia*: LaPolla et al. 2010: 127.

#### Materials

- **Type status:** Syntype. **Occurrence:** recordedBy: A. Forel; individualCount: 1; sex: queen; **Location:** country: India; stateProvince: Uttarakhand; verbatimLocality: Dehradun; **Record Level:** institutionCode: MHNG, Geneva, Switzerland; collectionCode: CASENT0911010
- **Type status:** Other material. **Occurrence:** recordedBy: Aijaz A. Wachkoo; individualCount: 13; sex: workers; **Location:** country: India; stateProvince: Himachal Pradesh; verbatimLocality: Andretta; verbatimElevation: 930 m; decimalLatitude: 32.0744; decimalLongitude: 76.5856; **Event:** eventDate: Jun-21-2010; **Record Level:** institutionCode: PUPAC
- **Type status:** Other material. **Occurrence:** recordedBy: Aijaz A. Wachkoo; individualCount: 110; sex: workers; **Location:** country: India; stateProvince: Himachal Pradesh; verbatimLocality: Baijnath; verbatimElevation: 1125 m; decimalLatitude: 32.0527; decimalLongitude: 76.6500; **Event:** eventDate: Jun-17-2010; **Record Level:** institutionCode: PUPAC
- **Type status:** Other material. **Occurrence:** recordedBy: Aijaz A. Wachkoo; individualCount: 1; sex: worker; **Location:** country: India; stateProvince: Himachal Pradesh; verbatimLocality: Bakhra; verbatimElevation: 650 m; decimalLatitude: 31.4087; decimalLongitude: 76.4327; **Event:** eventDate: Oct-07-2008; **Record Level:** institutionCode: PUPAC
- **Type status:** Other material. **Occurrence:** recordedBy: Aijaz A. Wachkoo; individualCount: 99; sex: workers; **Location:** country: India; stateProvince: Himachal Pradesh; verbatimLocality: Bilaspur; verbatimElevation: 520 m; decimalLatitude: 31.3423; decimalLongitude: 76.7616; **Event:** eventDate: Jul-01-2010; **Record Level:** institutionCode: PUPAC
- **Type status:** Other material. **Occurrence:** recordedBy: Aijaz A. Wachkoo; individualCount: 204; sex: workers; **Location:** country: India; stateProvince: Himachal Pradesh; verbatimLocality: Chanaur; verbatimElevation: 600 m; decimalLatitude: 31.9067; decimalLongitude: 76.1428; **Event:** eventDate: Oct-20-2008; **Record Level:** institutionCode: PUPAC
- **Type status:** Other material. **Occurrence:** recordedBy: Aijaz A. Wachkoo; individualCount: 1; sex: worker; **Location:** country: India; stateProvince: Himachal Pradesh; verbatimLocality: Chohal; verbatimElevation: 450 m; decimalLatitude: 31.6666; decimalLongitude: 76.0666; **Event:** eventDate: Oct-08-2008; **Record Level:** institutionCode: PUPAC
- **Type status:** Other material. **Occurrence:** recordedBy: Aijaz A. Wachkoo; individualCount: 19; sex: workers; **Location:** country: India; stateProvince: Himachal Pradesh; verbatimLocality: Kotla; verbatimElevation: 500 m; decimalLatitude: 31.8821; decimalLongitude: 75.9963; **Event:** eventDate: Nov-30-2009; **Record Level:** institutionCode: PUPAC
- **Type status:** Other material. **Occurrence:** recordedBy: Aijaz A. Wachkoo; individualCount: 9; sex: workers; **Location:** country: India; stateProvince: Himachal Pradesh; verbatimLocality: Kotla; verbatimElevation: 500 m; decimalLatitude: 31.8821; decimalLongitude: 75.9963; **Event:** eventDate: Jul-13-2010; **Record Level:** institutionCode: PUPAC
- **Type status:** Other material. **Occurrence:** recordedBy: Aijaz A. Wachkoo; individualCount: 20; sex: workers; **Location:** country: India; stateProvince: Himachal Pradesh; verbatimLocality: Lwasa; verbatimElevation: 1200 m; decimalLatitude: 30.7394; decimalLongitude: 77.1528; **Event:** eventDate: Aug-27-2009; **Record Level:** institutionCode: PUPAC
- **Type status:** Other material. **Occurrence:** recordedBy: Aijaz A. Wachkoo; individualCount: 6; sex: workers; **Location:** country: India; stateProvince: Himachal Pradesh; verbatimLocality: Nagabari; verbatimElevation: 420 m; decimalLatitude: 32.3004; decimalLongitude: 75.8901; **Event:** eventDate: Jun-18-2009; **Record Level:** institutionCode: PUPAC
- **Type status:** Other material. **Occurrence:** recordedBy: Aijaz A. Wachkoo; individualCount: 3; sex: workers; **Location:** country: India; stateProvince: Himachal Pradesh; verbatimLocality: Nahan; verbatimElevation: 760 m; decimalLatitude: 30.5596; decimalLongitude: 77.2960; **Event:** eventDate: Sep-27-2009; **Record Level:** institutionCode: PUPAC
- **Type status:** Other material. **Occurrence:** recordedBy: Aijaz A. Wachkoo; individualCount: 216; sex: workers; **Location:** country: India; stateProvince: Himachal Pradesh; verbatimLocality: Poanta Sahib; verbatimElevation: 420 m; decimalLatitude: 30.4384; decimalLongitude: 77.6239; **Event:** eventDate: May-09-2009; **Record Level:** institutionCode: PUPAC
- **Type status:** Other material. **Occurrence:** recordedBy: Aijaz A. Wachkoo; individualCount: 171; sex: workers; **Location:** country: India; stateProvince: Himachal Pradesh; verbatimLocality: Poanta Sahib; verbatimElevation: 420 m; decimalLatitude: 30.4384; decimalLongitude: 77.6239; **Event:** eventDate: Aug-19-2009; **Record Level:** institutionCode: PUPAC
- **Type status:** Other material. **Occurrence:** recordedBy: Aijaz A. Wachkoo; individualCount: 8; sex: workers; **Location:** country: India; stateProvince: Himachal Pradesh; verbatimLocality: Renuka; verbatimElevation: 600 m; decimalLatitude: 30.6083; decimalLongitude: 77.4615; **Event:** eventDate: Aug-26-2009; **Record Level:** institutionCode: PUPAC
- **Type status:** Other material. **Occurrence:** recordedBy: Aijaz A. Wachkoo; individualCount: 5; sex: workers; **Location:** country: India; stateProvince: Himachal Pradesh; verbatimLocality: Terrace; verbatimElevation: 430 m; decimalLatitude: 31.9234; decimalLongitude: 75.9294; **Event:** eventDate: Jun-13-2009; **Record Level:** institutionCode: PUPAC
- **Type status:** Other material. **Occurrence:** recordedBy: Aijaz A. Wachkoo; individualCount: 2; sex: workers; **Location:** country: India; stateProvince: Himachal Pradesh; verbatimLocality: Terrace; verbatimElevation: 430 m; decimalLatitude: 31.9234; decimalLongitude: 75.9294; **Event:** eventDate: Sep-24-2009; **Record Level:** institutionCode: PUPAC
- **Type status:** Other material. **Occurrence:** recordedBy: Aijaz A. Wachkoo; individualCount: 4; sex: workers; **Location:** country: India; stateProvince: Himachal Pradesh; verbatimLocality: Terrace; verbatimElevation: 430 m; decimalLatitude: 31.9234; decimalLongitude: 75.9294; **Event:** eventDate: Jul-19-2010; **Record Level:** institutionCode: PUPAC
- **Type status:** Other material. **Occurrence:** recordedBy: Aijaz A. Wachkoo; individualCount: 3; sex: workers; **Location:** country: India; stateProvince: Jammu and Kashmir; verbatimLocality: Mansar; verbatimElevation: 690 m; decimalLatitude: 32.6979; decimalLongitude: 75.1489; **Event:** eventDate: Jul-13-2009; **Record Level:** institutionCode: PUPAC
- **Type status:** Other material. **Occurrence:** recordedBy: Aijaz A. Wachkoo; individualCount: 12; sex: workers; **Location:** country: India; stateProvince: Jammu and Kashmir; verbatimLocality: Surinsar; verbatimElevation: 700 m; decimalLatitude: 32.7009; decimalLongitude: 75.1512; **Event:** eventDate: Jul-14-2009; **Record Level:** institutionCode: PUPAC
- **Type status:** Other material. **Occurrence:** recordedBy: Aijaz A. Wachkoo; individualCount: 204; sex: workers; **Location:** country: India; stateProvince: Uttarakhand; verbatimLocality: Assan Barrage; verbatimElevation: 750 m; decimalLatitude: 30.4417; decimalLongitude: 77.6754; **Event:** eventDate: Jun-30-2010; **Record Level:** institutionCode: PUPAC
- **Type status:** Other material. **Occurrence:** recordedBy: Aijaz A. Wachkoo; individualCount: 11; sex: workers; **Location:** country: India; stateProvince: Uttarakhand; verbatimLocality: Dakpathar; verbatimElevation: 750 m; decimalLatitude: 30.5164; decimalLongitude: 77.7848; **Event:** eventDate: Aug-20-2009; **Record Level:** institutionCode: PUPAC
- **Type status:** Other material. **Occurrence:** recordedBy: Aijaz A. Wachkoo; individualCount: 12; sex: workers; **Location:** country: India; stateProvince: Uttarakhand; verbatimLocality: Forest Research Institute, Dehradun; verbatimElevation: 640 m; decimalLatitude: 30.3416; decimalLongitude: 77.9903; **Event:** eventDate: Sep-30-2008; **Record Level:** institutionCode: PUPAC
- **Type status:** Other material. **Occurrence:** recordedBy: Aijaz A. Wachkoo; individualCount: 1; sex: worker; **Location:** country: India; stateProvince: Uttarakhand; verbatimLocality: Forest Research Institute, Dehradun; verbatimElevation: 640 m; decimalLatitude: 30.3416; decimalLongitude: 77.9903; **Event:** eventDate: Oct-02-2008; **Record Level:** institutionCode: PUPAC
- **Type status:** Other material. **Occurrence:** recordedBy: Aijaz A. Wachkoo; individualCount: 16; sex: workers; **Location:** country: India; stateProvince: Uttarakhand; verbatimLocality: Forest Research Institute, Dehradun; verbatimElevation: 640 m; decimalLatitude: 30.3416; decimalLongitude: 77.9903; **Event:** eventDate: Jul-31-2009; **Record Level:** institutionCode: PUPAC
- **Type status:** Other material. **Occurrence:** recordedBy: Aijaz A. Wachkoo; individualCount: 5; sex: workers; **Location:** country: India; stateProvince: Uttarakhand; verbatimLocality: Rajaji Forest Area, Dehradun; verbatimElevation: 660 m; decimalLatitude: 30.2483; decimalLongitude: 77.9878; **Event:** eventDate: Aug-05-2009; **Record Level:** institutionCode: PUPAC
- **Type status:** Other material. **Occurrence:** recordedBy: Aijaz A. Wachkoo; individualCount: 1; sex: queen; **Location:** country: India; stateProvince: Uttarakhand; verbatimLocality: Rajaji Forest Area, Dehradun; verbatimElevation: 660 m; decimalLatitude: 30.2483; decimalLongitude: 77.9878; **Event:** eventDate: Aug-05-2009; **Record Level:** institutionCode: PUPAC
- **Type status:** Other material. **Occurrence:** recordedBy: Aijaz A. Wachkoo; individualCount: 9; sex: males; **Location:** country: India; stateProvince: Uttarakhand; verbatimLocality: Rajaji Forest Area, Dehradun; verbatimElevation: 660 m; decimalLatitude: 30.2483; decimalLongitude: 77.9878; **Event:** eventDate: Aug-05-2009; **Record Level:** institutionCode: PUPAC
- **Type status:** Other material. **Occurrence:** recordedBy: Aijaz A. Wachkoo; individualCount: 21; sex: workers; **Location:** country: India; stateProvince: Uttarakhand; verbatimLocality: Rajaji Forest Area, Dehradun; verbatimElevation: 660 m; decimalLatitude: 30.2483; decimalLongitude: 77.9878; **Event:** eventDate: Aug-11-2009; **Record Level:** institutionCode: PUPAC
- **Type status:** Other material. **Occurrence:** recordedBy: Aijaz A. Wachkoo; individualCount: 18; sex: workers; **Location:** country: India; stateProvince: Uttarakhand; verbatimLocality: Rajaji Forest Area, Dehradun; verbatimElevation: 660 m; decimalLatitude: 30.2483; decimalLongitude: 77.9878; **Event:** eventDate: Sep-07-2010; **Record Level:** institutionCode: PUPAC
- **Type status:** Other material. **Occurrence:** recordedBy: Aijaz A. Wachkoo; individualCount: 7; sex: workers; **Location:** country: India; stateProvince: Uttarakhand; verbatimLocality: Selaqui, Dehradun; verbatimElevation: 670 m; decimalLatitude: 30.3720; decimalLongitude: 77.8605; **Event:** eventDate: Aug-08-2009; **Record Level:** institutionCode: PUPAC

#### Description

**Worker** (Fig. 1).

Measurements: HL 0.60–0.68; HW 0.47–0.56; EL 0.14–0.19; SL 0.70–0.77; PW 0.37–0.43; PrFL 0.56–0.64; PrFW 0.13–0.17; WL 0.73–0.88. Indices: CI 77.68–82.26; SI 137.25–151.16; REL 23.64–27.42 (n = 8).

Head broadly oval; distinctly longer than wide, slightly wider posteriorly, lateral margins convex, posterior margin shallowly concave to gently convex with rounded posterolateral corners. Clypeus subcarinate in the middle; anterior clypeal margin weakly concave. Mandibles with six teeth. Eyes oval, weakly convex, covering one-third of lateral cephalic margin; three small ocelli present. Antennae long, scapes surpass the posterior margin by two-fifths their length.

Metanotal groove strongly developed, in lateral view interrupts the regular promesonotal convexity from propodeum; metanotal area long. Dorsal face of propodeal not higher than remainder of the notum, gently rounded; declivity steep.

In lateral view, petiole triangular with dorsum of the petiole well below the dorsum of propodeum, rounded above with posterior face much longer than anterior face.

Overall cuticle very smooth and shiny. Pubescence on body very sparse; almost entirely absent. Scapes and legs covered with abundant erect setae, and a very fine layer of pubescence. Erect setae of varying length cover head, pronotum, mesonotum and gaster. Setae very densely spaced on head and gaster.

Head brown; gaster brown to black; mesosoma, antennae and legs yellowish brown.

**Queen** (Fig. 2).

Measurements: HL 0.84; HW 0.80; EL 0.30; SL 0.93; PrFL 0.82; PrFW 0.23; WL 0.88. Indices: CI 95.59; SI 115.82; REL 35.71 (n = 1).

Generally matches worker description, with modifications expected for caste and the following differences: Body covered with dense pubescence and fine punctulae. Head subtriangular, posterior margin concave; in lateral view, petiole with flat dorsum. Head yellow brown, gaster brown, a shade lighter than in workers.

**Male** (Fig. 3).

Measurements: HL 0.52–0.55; HW 0.42–0.46; EL 0.22–0.24; SL 0.66–0.72; PrFL 0.56–0.62; PrFW 0.13–0.15; WL 0.75–0.85. Indices: CI 80.00–85.42; SI 146.34–157.89; REL 41.67–44.44 (n = 8).

Head broadly oval; longer than wide; eyes large, subglobulose, projecting beyond head outline in full-face view; three prominent ocelli present. Antennae 13 segmented, filiform, scapes long, surpass posterior margin by about half their length. Mandibles slender, curved strap like with prominent, pointed apical and a small preapical tooth, remainder of masticatory margin smooth, without any teeth or denticles. Basal angle rounded, indistinct and seamlessly blends into inner mandibular margin.

Mesosoma enlarged to accommodate flight muscles; in lateral view scutum and scutellum flat; propodeum indistinct, lower than remainder of notum with very short dorsal face and long declivitous face.

Petiole as in worker; gaster elongated.

Parameres paddle-shaped, rounded apically, turning strongly inward toward midline of body posteriorly, as long as digiti; long setae extending off of parameres. Cuspi long and tubular reaching digiti dorsally; bent toward digiti; digiti weakly anvil-shaped; directed upward and covered with short peg-like teeth; digiti visible in lateral view. Penis valves projecting barely past parameres.

Sculpture, vestiture and color as in worker caste.

#### Distribution

This species is general in distribution in low altitude areas of Northwest India. Nests can be found easily underneath stones and leaf litter whilst foraging workers are fairly frequent on trees and fallen trunks.

#### Notes

The worker caste of this species is described here for the very first time and can be easily distinguished by the oval shape of head, and smooth and shiny cuticle without any pubescence. *Nylanderia smythiesii* although, abundant in its type locality, the Shivalik range of Northwest Himalaya, its worker caste was undescribed to date. Type locality, images of syntype queen examined on AntWeb (www.antweb.org): CASENT0911010, the description of female and male castes and the line drawings of external genitalia by Forel (1894) are all in conformity with our specimens and therefore, render us reasonable to describe its worker caste.

### Nylanderia assimilis

(Jerdon, 1851)

Formica assimilis  Jerdon, 1851 – Jerdon 1851: 125 (w.) INDIA. Combination in *Prenolepis*: Dalla Torre 1893: 177; in Paratrechina (Nylanderia): Emery 1925: 219; in *Nylanderia*: LaPolla et al. 2010: 127. **Species inquirenda**

#### Notes

The original descriptions is exceedingly inadequate and very little is known about the collection of Jerdon. Dalla Torre (1893) transferred it from *Formica* to *Prenolepis*, possibly because the body size, presence of large eyes and scattered white hairs all over the body mentioned in description mostly match the generic diagnosis of *Prenolepis*; subsequently Emery (1925) by implication transferred it to *Nylanderia*. Most of the taxa by Jerdon are invalid and have been synonymized or treated as *incertae sedis*. From the original description it could easily be argued that *Nylanderia assimilis* is yet another synonym of *Paratrechina longicornis* (Latreille, 1802), as the combination of characters mentioned by Jerdon (1851): large eyes, white hairs, body color and size are present only in *Paratrechina longicornis* of *Prenolepis* genus group from India. Moreover, Jerdon (1851) mentions it to be a frequent visitor of flowers, which also agrees with opportunistic feeding behavior of *Paratrechina longicornis*. Further, a Ph.D. thesis has been done on Formicidae of Malabar, India, the type locality of *Nylanderia assimilis*, but it could not be found. Bingham (1903) also excluded it from his study. Therefore, authors argue for its doubtful status and consider it *species inquirenda*.

## Identification Keys

### Key to Indian species of *Nylanderia* (workers)

**Table:** 

1	Head and mesosomal dorsum covered with a dense network of microreticulate sculpture	***Nylanderia birmana* (Forel)**
–	Head and mesosomal dorsum not microreticulate, either smooth or covered with fine punctulae	2
2	Gastral dorsum without a layer of pubescence underneath erect setae	***Nylanderia smythiesii* (Forel)**
–	Gastral dorsum with a layer of pubescence underneath erect setae	3
3	Head oval; scapes relatively shorter, surpass posterior margin of head by ≤ 1/3^rd^ their length	***Nylanderia taylori* (Forel)**
–	Head subquadrate; scapes long, surpass posterior margin of head by ≥ 2/5^th^ their length	4
4	Compound eye small, distinctly < 1/3^rd^ the length of lateral cephalic margin	***Nylanderia indica* (Forel)**
–	Compound eye large, ≥ 1/3^rd^ the length of lateral cephalic margin	5
5	Body unicolorous brown to black	***Nylanderia bourbonica* (Forel)**
–	Body distinctly bicoloured, with yellow brown to reddish brown mesosoma, and darker brown gaster	***Nylanderia yerburyi* (Forel)**

## Supplementary Material

XML Treatment for Nylanderia smythiesii

XML Treatment for Nylanderia assimilis

## Figures and Tables

**Figure 1a.**
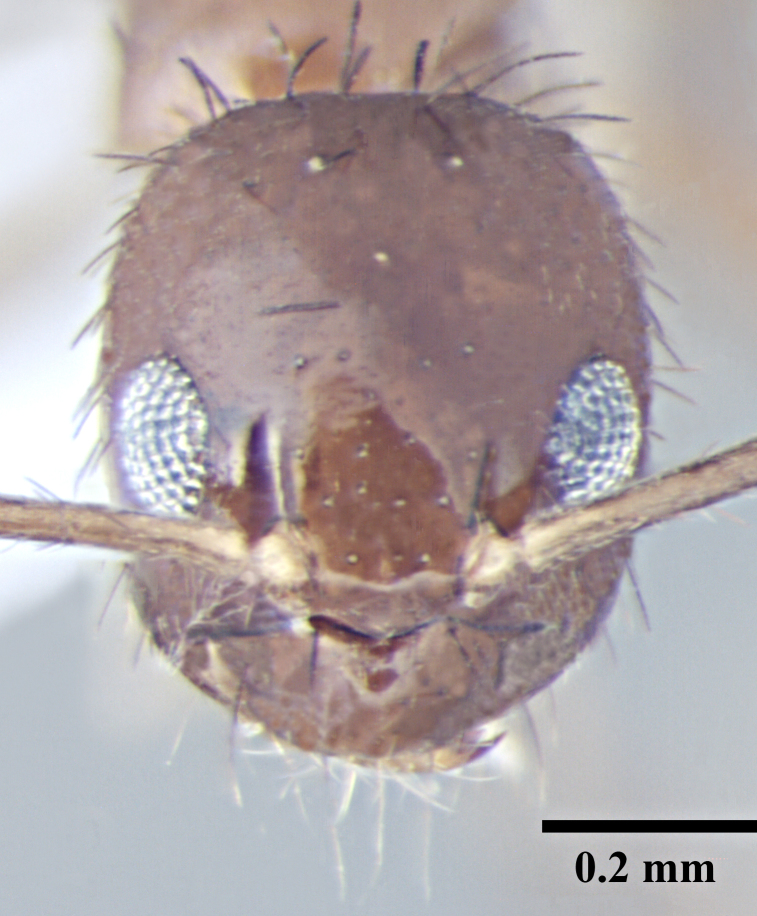
Worker Head, full face view

**Figure 1b.**
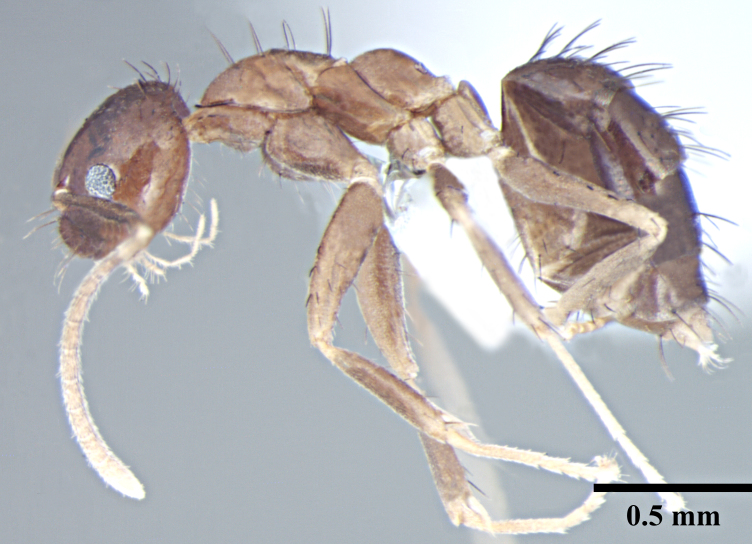
Worker Body, lateral view

**Figure 1c.**
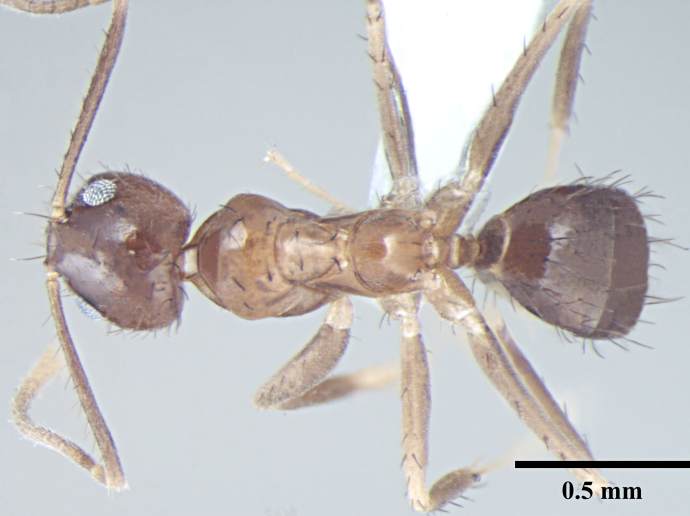
Worker Body, dorsal view

**Figure 2a.**
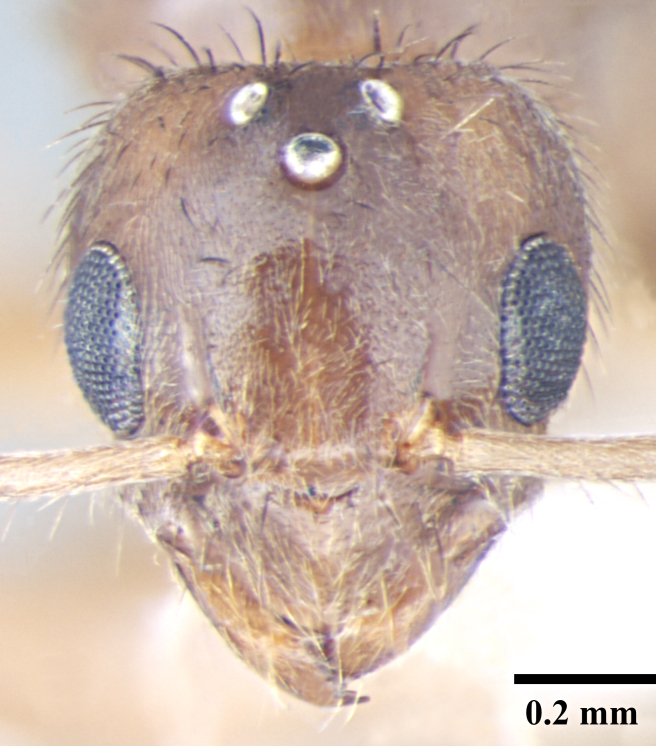
Queen Head, full face view

**Figure 2b.**
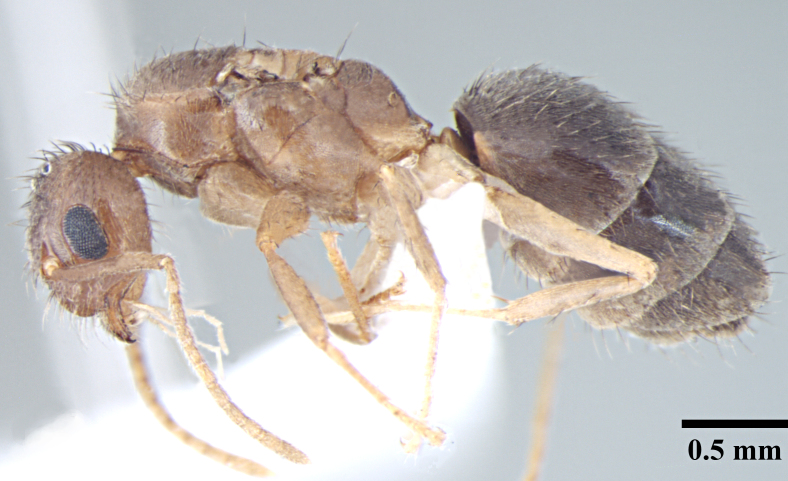
Queen Body, lateral view

**Figure 2c.**
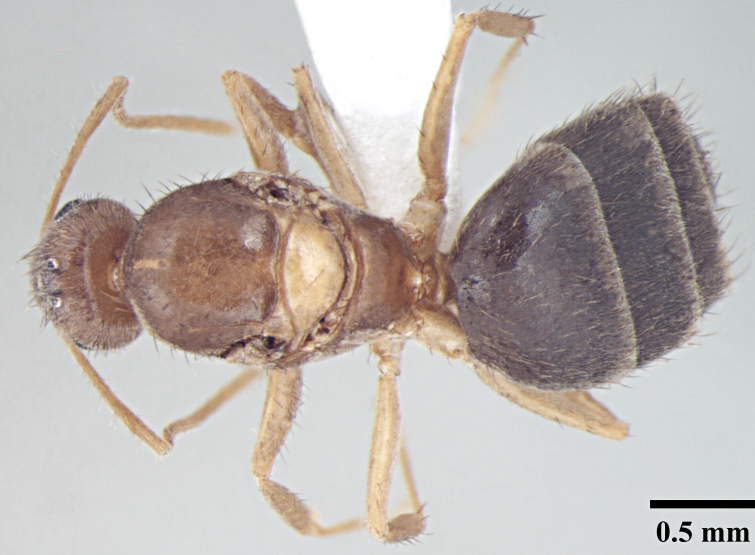
Queen Body, dorsal view

**Figure 3a.**
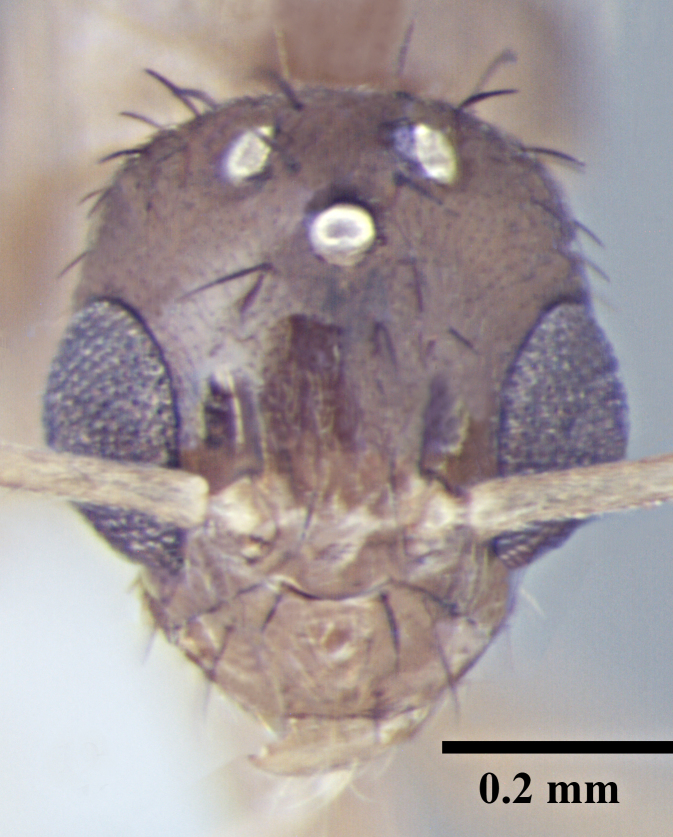
Male Head, full face view

**Figure 3b.**
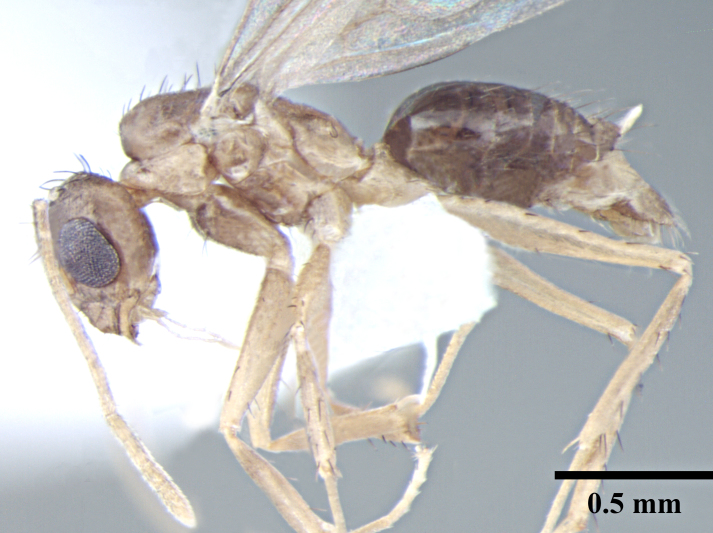
Male Body, lateral view

**Figure 3c.**
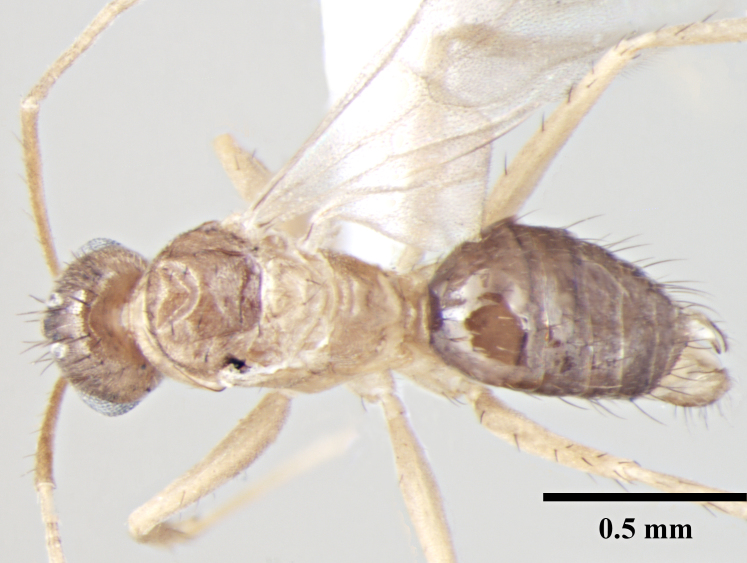
Male Body, dorsal view
